# True Cost Accounting of a healthy and sustainable diet in Italy

**DOI:** 10.3389/fnut.2022.974768

**Published:** 2022-07-29

**Authors:** Bianca Minotti, Marta Antonelli, Katarzyna Dembska, Davide Marino, Gabriele Riccardi, Marilena Vitale, Ilaria Calabrese, Francesca Recanati, Annalisa Giosuè

**Affiliations:** ^1^Department of Bioscience and Territory, University of Molise, Campobasso, Italy; ^2^Department of Humanities, Faculty of Economics and Management, Czech University of Life Science, Prague, Czechia; ^3^Barilla Center for Food & Nutrition Foundation, Parma, Italy; ^4^Division on Climate Change, Impacts on Agriculture, Forests and Ecosystem Services (IAFES), Fondazione Centro Euro-Mediterraneo sui Cambiamenti Climatici, Viterbo, Italy; ^5^Department of Clinical Medicine and Surgery, University of Naples “Federico II”, Naples, Italy

**Keywords:** TCA, sustainable diets, healthy diets, carbon footprint, water footprint

## Abstract

It is widely upheld that global food systems are unsustainable. Sustainable diets are gaining prominence as key components to entangle global food system challenges, as well as to transition towards the pathway of the 2030 Agenda and the Sustainable Development Goals (SDGs). Hence, sustainable and healthy diets are at the core of much research with the aim to bring together nutritional adequacy, cultural acceptability, environmental sustainability, economic affordability, and shape future consumption patterns. This article contributes to advancing knowledge on sustainable diets by proposing a True Cost Accounting method to assess the cost and impact of the adoption of a more sustainable and healthier diet, using Italy as an illustration. The research analyses the complexity of a diet from an environmental, health, and socioeconomic point of view and defines a new assessment framework that can be replicated and adapted to other contexts. Results show that in Italy, the adoption of a sustainable and healthy diet has a 47% lower carbon footprint and 25% lower water footprint than the current diet, while impacting 13% less on the average income and food monthly expenditure. Also, the desirable diet has a 21% lower impact on the sanitary costs related to cardiovascular disease. This study corroborates that the consumption of the desirable diet would provide a total cost saving of 741 EUR per year per capita, if we consider its impact on the environment, health, and socio-economic costs.

## Introduction

Evidence of the need for a paradigm shift from current global diets for their negative impact on the environment and health is thriving ((1); Food and Agriculture Organization of the United Nations (FAO), (2–11)). Dietary patterns changed dramatically in the past 50 years representing a threat to the health and well-being of the population and environment (12). Unhealthy diets—low in fruits, vegetables, and whole grains and high in red and processed meat, sugar, and sodium—are now one of the top risk factors for premature mortality and diseases (13). Of note, as much as 50% of total cardiovascular diseases (CVDs) occurring in Europe are attributable to food choices (14); CVD, and, in particular, coronary heart disease (CHD) (i.e., myocardial infarction and other ischemic heart diseases) are the leading cause of death and disability not only in Europe, but also in Italy. Many studies (5, 6, 8, 10, 15, 16) have shown that healthy and sustainable diets can reduce wildlife loss, premature deaths, food-related greenhouse gas emissions (GHGs), and poverty along with improving social inclusion, biodiversity, fair trade, individual health, and many other “wicked” issues (17). Sustainable diets are, indeed, a very complex topic, as they attempt to be nutritionally adequate, culturally acceptable, economically affordable, and have a low environmental impact. Hence, designing healthy and sustainable diets changes according to the way problems and solutions are defined.

True Cost Accounting (TCA) can be a way to assess different types of costs and impacts, including various aspects of the matrix that composes the food system. TCA is “a critical tool to help us, as a global community, better understand the impacts of food systems, address the most harmful practices, and find new, positive pathways forward” (18). The concept emerged from the increased awareness of the negative externalities of food production and the food system supply chain, first mentioned in a 2009 TIME Magazine cover article, “The Real Cost of Cheap Food” by Bryan Walsh. Since that moment, TCA has been used in many ways (19) and with many scopes, but all the assessments follow the same guiding principles (18), which refer to systemic and multilateral approaches that aim at transparency and participation, to achieve transformative governance and redirecting structural power toward food sovereignty and agro-ecological principals. Indeed, “by evaluating the impacts—both the positive and negative—inherent in different food systems and making these impacts transparent, decision-makers on farms and in governments, institutions, and businesses can make better-informed decisions that consider the economic, environmental, and social impacts of their choices” (18). As the Initiative on the True Value of Food announced in September 2021 by the United Nations during the Food System Summit, which created a community of experts “who stand ready to support country efforts to consider, trial, implement, and evaluate true cost, value, and price of food actions and policy change” (20); the topic is highly relevant. In the domain of diets, the TCA TEEBAgriFood Framework (19) has been applied in France for a dietary comparison, evaluating the welfare and sustainability effects of six diets and the twenty-two food groups (21), showing that healthy diets usually have environmental positive effects even though they are highly cost-effective. Peters et al. (22) also applied the TEEBAgriFood Framework performing a carrying capacity analysis of the US agricultural land in relation to ten different diets and land requirements. The research demonstrated that the carrying capacity is lower for vegetarian and vegan diets and higher for those containing animal products (meat or lacto-vegetarian diet).

Following the same framework, this study formulated a new model of TCA to assess the sustainability of a diet, comparing an ideal healthy and sustainable diet, with an illustration for Italy. Although the study by Conforti and D’Amicis (23) shows that Italy in the first decade of this century has moved toward healthier eating habits; consumption of red meat and animal fat is still too high, while that of consumption of vegetables and whole grains is rather low (23, 24). These dietary characteristics are known to be related to chronic disease incidence (25). Indeed, De Marco et al. (25) show that adherence to the traditional Mediterranean diet has decreased by 56% from 1961 to 2007 in the European Mediterranean countries, where the population fails to meet dietary recommendations with excessive saturated fatty acids, added sugars and sodium, and reduced fiber consumption, resulting in a negative impact on health, water consumption, and ecological footprint. However, the country results be an interesting case study also because of the traditional affinity with the Mediterranean diet, which is considered an example of a healthy and sustainable diet (10, 25–27), promoting high healthy life expectancy and the relatively low GHGs compared to other European countries (28).

This article will first describe the Materials and Methods used and then highlight the main findings and results. Then, a brief conclusion is drawn, leaving space for discussion and presentation of possible further research.

## Materials and methods

This study compares a healthy and sustainable diet (desirable diet) with the current diet of Italians (current Italian diet) in terms of environmental, health, and socio-economic impact, using the True Cost Accounting framework.

The procedure to define the desirable diet has been extensively described elsewhere (29). In brief, a systematic review of the literature was carried out, searching for evidence linking the consumption of individual foods/food groups to the risk of CHD in prospective studies as summarized in the available meta-analyses. The most common foods utilized worldwide were grouped according to their specific features and nutritional properties (i.e., processed meat, red meat, white meat, animal fat and tropical vegetable oils, fish, eggs, refined cereals with high glycemic index and potatoes, refined cereals with low glycemic index, whole-grain cereal foods, legumes, nuts, non-tropical vegetable oils, fresh fruit, vegetables).

The association between the consumption of each food group/item and cardiovascular outcomes was evaluated by comparing the relative risk and CIs between the highest and the lowest consumption group. Data from dose–response analyses were used to identify the amounts of foods with the strongest association with the outcomes or in the absence of a statistically significant association, the thresholds of intake above which data do not allow to exclude an increased CHD risk. For each food group/item, the most updated and comprehensive dose-response meta-analysis was chosen as a reference among the available ones.

The current diet was provided by an adaptation of Vitale et al. (27) study in which the Italian diet is calculated using the FAOSTAT Food Balance Sheets (FBSs) (30). This study extracted the food supply “as the sum of the total quantity of foodstuffs produced (including production, imports, and stock changes) minus exports, food use other than human feed, and food losses during food transport, storage, and processing” (27). The potential food waste was also subtracted from the food supply to obtain the actual food consumption data. Finally, the Italian per day per capita diet was obtained by dividing the quantity of each food group selected by the population (27). Of note, Vitale et al. (27) have been used as a starting point regarding the amount of foods available for consumption and the following TCA calculations have used a methodology specific to this study (more on Supplementary material section “Explanation methodology”). The food groups utilized are those of Vitale et al. (27) crossed with a mapping of the groups of the desirable diet for the optimization of CHD prevention.

Table 1 shows the average portions per day for each food group in both the diets. The consumption of extra products such as alcohol, coffee, snacks, cakes, pastries, and other similar products is not considered in this study.

**TABLE 1:** Daily average consumption of the various food groups in the desirable and the current Italian diet (Source: Authors).

Food group	Current Italian Diet (g/day)	Desirable diet (g/day)
Fruit	181	400
Vegetables	205	400
Legumes	12	46
Nuts	18	30
Refined cereals	288	94
Potatoes	50	50
Wholegrain	27	150
Beef and pork	124	14
Poultry	41	43
Fish	57	103
Eggs	28	43
Dairy products	571	329
(milk, cheese and yogurt)		
Animal fat and tropical vegetable oils	14	3
Non-tropical vegetable oils	46	25−40
Sugars	79	20

**FIGURE 1:**
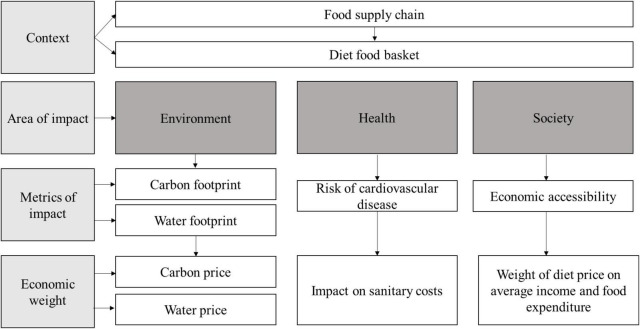
TCA model (Source: Authors).

This study assessed the true cost of both the diets from an environmental, health, and socioeconomic point of view. As the main reference to build the TCA model, the research referred to the TEEBAgriFood Framework, an evaluation framework that helps identify all the invisible costs and benefits from natural, human, social, and produced capital (19). For the aim of this research, the TEEBAgriFood Framework has been used as a theoretical framework to create a new model of TCA developed in this study and is shown in Figure 1. Indeed, the TCA model formulated in this study rotates around impact rather than capital to understand the influence that a diet has on a given societal environment, which is a strong difference between the TEEBAgriFood Framework and the model developed.

The model aims at unifying under the true cost umbrella different indicators and data regarding the environmental impact, the impact on health, and the socio-economic impact of the two diets selected. The idea is to provide a model that uses existing data sources (open access or accessible on request) to make it replicable for other case studies.

The impacts on the three areas considered are calculated with different indicators and then weighted from an economic point of view. As shown in Figure 1, for each impact, a measure has been selected to exemplify the influence of the diet on a specific area of interest and an economic weight has been added to assign an economic cost to each impact. To achieve results that could be comparable and coherent among the three impacts analyzed, the two assessed diets have been represented by considering a specific food basket, including food items that were available in all the databases selected (more on Supplementary material section “Explanation methodology”). It is important to highlight that the study did not consider all the externalities, but selected one parameter for each of the three pillars of sustainability: environmental, economic, and social.

For each area of analysis, different databases have been considered and calculations have been done:

(1). Environment
  First, data relating to each food item were found within the database provided by Petersson et al. (31). All the data on CO_2_ emissions and water consumption for each specific food was multiplied for the daily average consumption obtaining the quantity of CO_2_eq generated and the quantity of water consumed by each assessed diet. Then, the economic cost of CO_2_ emission was calculated using the Carbon Price Viewer from Sandbag^1^, as the official source by the Emission Trading System (ETS) of the carbon price fluctuation at the European level and the economic cost of water consumption has been obtained through the average Italian tariffs of tap irrigation water (32).
(2). Health
  The health cost of the desirable and the current Italian diet was assessed based on the Mediterranean Diet Score developed by Panagiotakos et al. (33, 34). This score incorporates the inherent characteristics of the Mediterranean dietary pattern in order to evaluate the nutritional status of an individual and to link his food choices with various health outcomes. The score ranges from 0 to 55 and higher values are indicative of higher adherence to the Mediterranean diet. It has been utilized to evaluate how much the diets of the populations resemble the features of the traditional Mediterranean diet. This study was used to estimate the impact of the diets on the development of CHD in the Italian population and on the relative costs for the health system, using the data by Roggeri et al. (35).
(3). Socioeconomic
  To assess the socioeconomic impact of both the diets, the weekly per capita cost of food groups was calculated using official data provided by the ISMEA food price database (more on Supplementary material section “Explanation methodology”). Each food in the food group was calculated by dividing Italian sales volumes (tonnes) and values (EUR) data at the national level and weighted according to daily average portions. Then, the monthly cost of the desirable diet and the current Italian diet was crossed with the ISTAT^2^ data on the average monthly income and expenditure of a family consisting of a single member. This helped formulate an index of economic affordability of the diet following Bernaschi and Marino (36) model. The affordability index (36) measures the distance between the real incidence of spending on a diet compared to the average values. The greater the distance between these two values is, the greater the difficulty is in accessing that diet. In this study, the index has been calculated as follows: first, the incidence of food expenditure on the average declared income was calculated. Then, the average incidence of food expenditure on consumption was calculated by dividing the average monthly food expenditure by the average monthly non-food expenditure of the ISTAT 2019 data (more on Supplementary material section “Explanation methodology”).

In this study, the TCA has been calculated from a weekly point of view; however, the model can be used for any type of timeframe according to the data availability.

## Results

**FIGURE 2:**
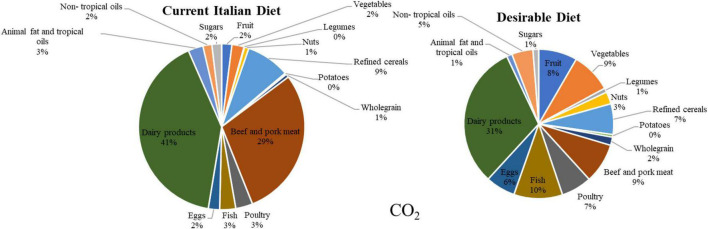
CO_2_ emissions associated with the weekly consumption of foods in the desirable and the current Italian diet (Source: Authors).

The results present an assessment of the TCA of diets in three areas: environment, health, and socio-economic. To evaluate the environmental cost, this study considered the carbon and water footprint of both the diets. The desirable diet shows a weekly production of 14.99 kg CO_2_, which accounts for a calculated cost of 0.82 EUR/kg per week [considering that the carbon price fluctuates around 0.05 EUR/kg (37)^3^]. The current diet causes around 28.52 kg CO_2_ per week, corresponding to 1.57 EUR of CO_2_ per week. These results show that the desirable diet causes 47% less CO_2_ emissions than the current diet. In particular, the two food groups that cause the highest emission of CO_2_ in the current diet are beef and pork meat together with dairy foods, accounting for 70% of the total CO_2_ production (Figure 2 and Table 2).

**TABLE 2:** Price for CO_2_ per week for the desirable and the current Italian diet (Source: Authors).

Food group	Current Italian diet	Desirable diet
Fruit	0,03 €	0,07 €
Vegetables	0,04 €	0,07 €
Legumes	0,00 €	0,01 €
Nuts	0,01 €	0,02 €
Refined cereals	0,13 €	0,05 €
Potatoes	0,005 €	0,005 €
Wholegrain	0,01 €	0,01 €
Beef and pork meat	0,46 €	0,07 €
Poultry	0,05 €	0,05 €
Fish	0,05 €	0,09 €
Eggs	0,03 €	0,05 €
Dairy products	0,64 €	0,26 €
Animal fat and tropical oils	0,05 €	0,01 €
Non- tropical oils	0,03 €	0,04 €
Sugars	0,03 €	0,01 €
TOT	1,57 €	0,82 €

**FIGURE 3:**
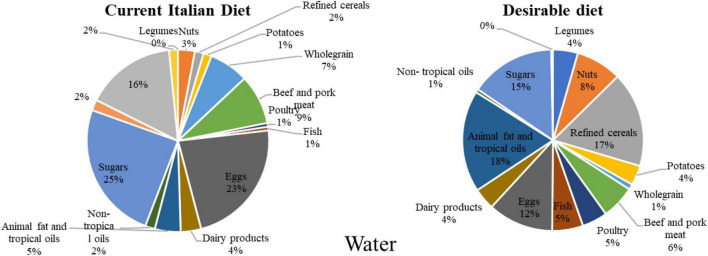
Water footprint associated with the weekly consumption of foods in the desirable and the current Italian diet (Source: Authors).

Regarding water consumption^4^, the desirable diet accounts for 21,812.58 liters of water per week, while the current diet is almost 29,000, liters per week. These results show that the desirable diet consumes 25% less water than the current diet. Again, beef and pork together with milk and dairy are the two groups accounting for most of the water use (13,693.09 liters of water per week). The economic cost of water consumption has been calculated by considering an average cost of 0.001 EUR/L (given that the average Italian fees for tap irrigation water are around 1.16 EUR/m^3^ and 0.80 EUR/m^3^) (32). Using this parameter, this study assessed an expenditure of 21.81 EUR for the 21,812.58 liters of water used by the desirable diet weekly and a total of 28.92 EUR for the 28,922.85 liters used by the current Italian diet (Figure 3 and Table 3).

**TABLE 3:** Cost of irrigating water per week due to food consumed in the desirable and the current Italian diet (Source: Authors).

Food group	Current Italian diet	Desirable diet
Fruit	0,86 €	1,91 €
Vegetables	0,45 €	0,88 €
Legumes	0,42 €	1,63 €
Nuts	2,03 €	3,38 €
Refined cereals	2,56 €	0,70 €
Potatoes	0,19 €	0,19 €
Wholegrain	0,19 €	1,20 €
Beef and pork meat	6,57 €	0,90 €
Poultry	1,05 €	1,10 €
Fish	1,32 €	2,33 €
Eggs	0,50 €	0,77 €
Dairy products	7,12 €	3,62 €
Animal fat and tropical oils	0,55 €	0,12 €
Non- tropical oils	4,64 €	3,03 €
Sugars	0,45 €	0,06 €
TOT	28,92 €	21,81 €

Moreover, from a nutritional perspective, the Mediterranean Diet Score (3, 33, 5) has been calculated based on the frequency of consumption of the various food groups for both the diets (Table 4).

**TABLE 4:** Frequency of consumption of the various food groups in the current diet and the desirable one and their impact on the Mediterranean Diet Score (Source: Authors).

Food group	Food frequency (servings/Month)		Mediterranean Diet Score	
	Current	Desirable	Current	Desirable
Wholegrain	1−4	> 18	1	5
Potatoes	1−4	1−4	1	1
Fruit	9−12	> 18	3	5
Vegetables	9−12	> 18	3	5
Legumes	5−8	> 18	2	5
Fish	13−18	> 18	4	5
Red Meat	> 18	1−4	0	4
Poultry	9−12	9−12	2	2
Dairy products	> 18	>18	0	0
Olive oil	Daily	Daily	5	5
		Total	21	37

For the current diet, the Mediterranean Diet Score of 21 is estimated to correspond to 10-year risk of 5.4% of coronary events, while for the desirable diet, the score is estimated to be 37, corresponding to 10-year risk of 4.3% (34). In particular, the desirable diet is characterized by higher food frequencies of whole grain, fruit, vegetables, legumes, and fish than the current diet. By implementing the desirable diet, the Italian adult population would experience a 21% reduction in the rate of CHD.

From the perspective of the Italian health system, this reduction would strongly impact the health costs for the treatment of patients experiencing an acute coronary event. Indeed, the average annual cost for this type of patient is 5.503 EUR, including hospitalization, diagnostic and therapeutic procedures, and drugs (35), which leads to a total expense by the health system of 7,227,843,811 EUR, since there are 1,313,437 Italian people with a previous coronary event; this corresponds to an annual cost for each Italian citizen of 121.37 EUR (being the total Italian population around 59,550,000 people). A 21% reduction of the event rate would induce a saving of 1,517,847,200 EUR per year for the Italian Health System corresponding to 25 EUR per capita per year.

**FIGURE 4:**
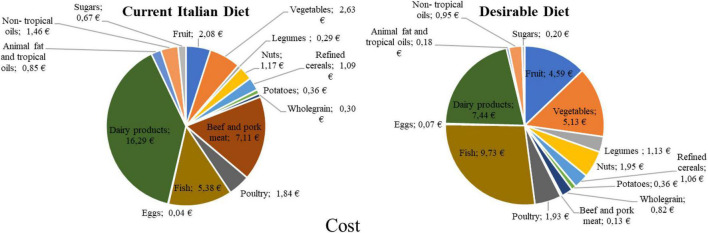
Weekly cost of foods consumed in the desirable and in the current Italian diet.

**FIGURE 5:**
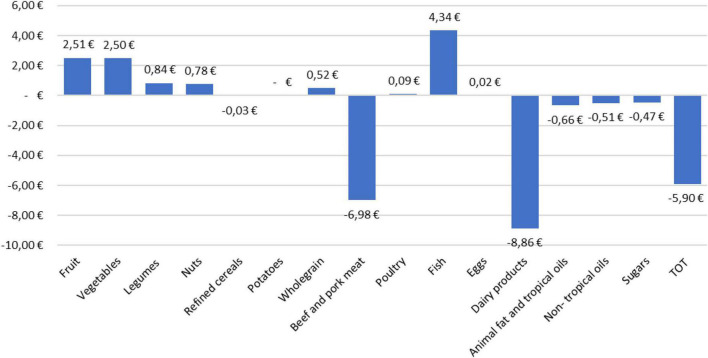
Changes in the expenditure for the various foods according to the desirable diet compared to the current Italian diet.

On the other side, from a consumer perspective, the desirable diet costs 35.67 EUR per week, while the current one costs 41.57 EUR per week. Looking at the weekly costs for the current and the desirable diet (Figure 4), the economic variance for every food group can be obtained by calculating the difference between the costs of the two diets. The results (Figure 4) show that each week, the desirable diet costs 5.9 EUR less than the current diet; this is mainly due to the reduced consumption of beef and pork meat and milk and dairy foods. The other food groups whose consumption in the desirable diet result less expensive than in the current one ar refined cereals, animal fat, non-tropical vegetable oils, and sugar. Overall, the desirable diet is 14% less expensive than the current Italian diet, especially because of the lower cost due to beef and pork and milk and dairy categories (Figure 5).

This study also calculated an affordability index (36) that crossed the average per capita income declared in Italy—1582.17 EUR per month and the average per capita food expenditure per month—187.04 EUR out of 1009.88 EUR of total per capita monthly expenditure.

**FIGURE 6:**
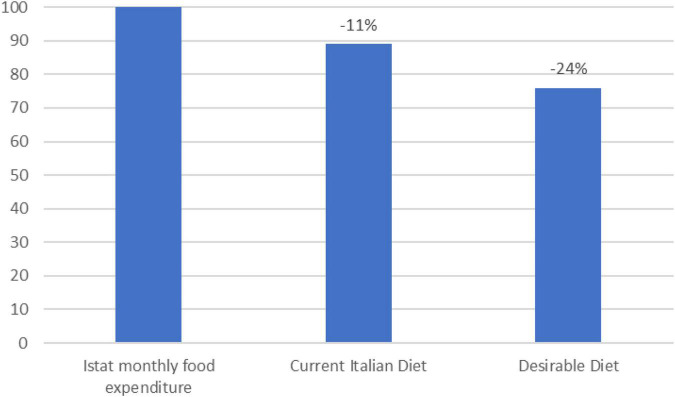
Affordability index of the desirable and the current Italian diet in reference to the ISTAT monthly food expenditure (2020) (Source: Authors).

The results show that the desirable diet is more economically accessible as it allows 24% savings on the monthly food expenditure, while the current diet is only 11% savings. Considering that this study used the same food basket for both the diets, the 13% variance (which results from the difference between -24% IA desirable diet and -11% IA current Italian diet) is attributable to the appropriate amount and frequency of consumption of the various foods rather than to the consumption of different food items between the two diets. Indeed, as shown in Figure 6, the distance between the desirable diet and the ISTAT reference data highlights the savings that this diet could provide. Conversely, the distance between the reference data and the current diet is mainly due to the different food baskets between the ISTAT and this study (see Supplementary material section “Explanation methodology”), but also to a substantial difference in frequency and amount of foods consumed compared to the desirable diet.

**FIGURE 7:**
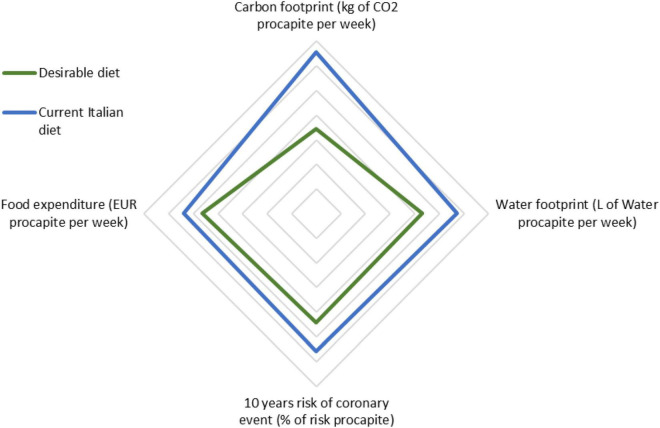
Multi-criteria comparison of the desirable and the current Italian diet based on the results of the study (Source: Authors). Figure 7 has been obtained by calculating the variance for the desirable and the current Italian diet from the average of the results for each variable. The values for each variable highlighted in the results have therefore been divided by the average (between the desirable and the current diet) and multiplied by 100 to obtain the variance showed.

Finally, Figure 7 summarizes the results of the study underlining the importance of a multi-criteria perspective, illustrating that the desirable diet performs better in all the considered dimensions and related criteria. Indeed, although economic accounting is important as it assigns a common unit of measurement to different variables, the TCA aims at accounting all the costs, including those that cannot be priced. TCA should, therefore, be calculated with a social perspective rather than only an economic, and this study demonstrates its applicability. The total cost is indeed composed of three dimensions: an economic one with a strong social connotation related to food access and food poverty; an environmental one strongly linked with social justice, climate change, energy market, and more; and a health one, which has a direct correlation with individual well-being and social welfare.

From a solely economic perspective, Table 5 shows the yearly costs of each diet per capita and the consequent saving related to the consumption of the desirable healthy and sustainable diet.

**TABLE 5:** Costs per year per capita for each diet and yearly savings from the desirable diet adoption (Source: Authors).

Costs per year per capita	Carbon emissions	Irrigation water utilization	Coronary heart disease	Food acquisition	Total
Current diet	81,64 €	1.503,84 €	121,37 €	2.161,64 €	3.868,49 €
Desirable diet	42,64 €	1.134,12 €	95,88 €	1.854,84 €	3.127,48€
Saving from desirable diet	39 €	369,71 €	25,49 €	306,80 €	741 €

## Discussion and conclusion

To sum up, this study performed the TCA on the comparison between a desirable healthy and sustainable diet and the current diet of the Italian population. The results show that the desirable diet has a lower environmental impact in terms of carbon (−47%) and water footprint (−25%) than the current diet and brings about an economic saving of 0.75 EUR of CO_2_ per week and 7.11 EUR per liters of irrigation water per week. Also, this study highlights that the recommended diet has a lower impact on CHD by 21%, which directly relates to an annual saving of 25 Euro per year for each Italian citizen. Considering that the cost for CHD represents about one-third of the cost of total CVD and assuming that the change in dietary habits would have a similar impact on total CVD as on CHD, we can hypothesize that by considering the impact of dietary changes on total CVD expenditure, the money saving would be around 75 Euros per capita per year. If we also included the costs for diabetes care and cancer, the economic savings would be even larger, given the influence of dietary habits on both these diseases, whose incidence would presumably be reduced by the desirable diet. Obviously, these are only speculations since reliable data on the relationship between the Mediterranean Dietary Score and these health outcomes are lacking.

Finally, thanks to the affordability index developed, it is possible to state that the recommended healthy and sustainable diet is also 5% more economically affordable than the current one, considering the average Italian income and the monthly food expenditure declared. The results of this study are relevant as the country is experiencing a nutrition transition away from the Mediterranean diet, with a dramatic increase in overweight, obesity, and CHD prevalence. Indeed, the study by Cavaliere et al. (38) highlighted the great inconsistency of the current Italians’ diet with the Mediterranean model, especially for meat consumption, which is the first cause of high CO_2_ emissions and water use. The results of this study confirm that healthy and environmentally friendly diets are not necessarily more expensive than conventional diets, as other studies showed (23, 24, 26). A study by Germani et al. (26) shows that there is a substantial difference in which the budget is allocated along the food groups, which is confirmed by this study. Moreover, Donati et al. (24) demonstrated that according to their model “the sustainable diet may lead to a 51% cut in CO_2_ emissions, 9% reduction in H_2_O consumption, and 26% less land needed to regenerate the resources compared to the current diet” (p. 54), which is high in meat and low in fiber, confirming the low environmental impact of healthy diets showed by the other studies previously mentioned. Cavalieri et al. (38) also demonstrated that considering the current diet, the Italian population would have to make little effort to reach sustainable dietary goals.

The present study confirmed that beef and pork meat and milk and dairy products are the two food groups with the highest ecological impact (i.e., the highest carbon footprint), the highest impact on CHD risk, and they are more expensive than the other food groups. However, it is to underline that the desirable diet does not exclude these food groups. The strength of this diet relies in fact on changing the frequency and the serving sizes of foods consumed rather than on the exclusion of specific foods, in line with the approach underlying the utilization of the Mediterranean Diet Score. Indeed, the consumption of fruits, vegetables, legumes, and nuts in the current diet is almost 50% lower than in the desirable diet, while the consumption of beef and pork meat, animal fat, and sugar is more than double. A major strength of the desirable diet in relation to its health value, besides its impact on CHD, is indeed the large variety of foods included, which provides the whole range of nutrients.

This study provides a new TCA methodology that could be relevant for the cost accounting of other dietary patterns. However, despite the use of a reproducible methodology, the results should be referred to the Italian region since the sustainability of the recommended diet relies also on the cultural acceptability of specific food items (10). Therefore, more studies should be implemented on other dietary patterns linked to different cultural and gastronomic backgrounds to be compared with the findings of the present study.

Moreover, even though the utilization of a diet score to estimate the impact on the health of food consumption has the advantage of overcoming the limitations of the single nutrient/food approach—not being able to account for what people really eat—it has also some limitations. For instance, the components of the index are equally weighted and similarly scored, which may not be accurate since not all the foods or food groups have the same effect on the investigated health outcomes.

The local and regional production dimensions should also be investigated as it can reduce the energy and pollution costs related to transportation, along with non-industrial agricultural and farming production methods (39). Finally, as previously stated, TCA models should be participatory and aim at transforming the governance systems and redirecting structural power. Hence, more research should be directed to improve this TCA model with the help of field work.

## Data availability statement

The original contributions presented in this study are included in the article/Supplementary material, further inquiries can be directed to the corresponding author.

## Author contributions

BM, DM, and MA developed the idea and the framework of the study. BM conducted the research and wrote the manuscript. KD, AG, MV, IC, and GR investigated the nutritional and health aspects. FR supervised the environmental calculations. DM and MA supervised the entire project. All authors discussed the results and contributed to the final manuscript.

## Footnotes

1
https://ember-climate.org/data/carbon-price-viewer/

2 Istat is the italian national institute for statistics, see https://www.istat.it/.

3 It is important to note that today there is a strong variability in prices among the different carbon markets (European, international, and free market) and that the evolution on this sector is destined to evolve quickly, as suggested by Cevallos et al. (40) and the periodic reports by Ecosystem Marketplace (41). Also, the study assume that all carbon is an externality without considering how some portions of these externalities might already be internalized in the cost of food. This research acknowledge that the carbon market reflects some of the environmental externalities related to food production but knowing how many of these allowances are actually considered is not possible in the framework of this study.

4 The water use impact is calculated only by the market price without considering the potential externalities related to the problem of water scarcity. The study acknowledges that the issue of the water resource in Italy is an important topic which however is not easy to address. The wide use of water into agricultural production, the great import of this resource both nationally and globally, and more, are important externalities that however this article will not include.

## References

[1] Linseisen J Bergström E Gafa L Gonzalez CA Thiébaut A Trichopoulou A Consumption of added fats and oils in the European Prospective Investigation into Cancer and Nutrition (EPIC) centres across 10 European countries as assessed by 24-hour dietary recalls. *Public Health Nutr.* (2002) 5:1227–42. 10.1079/PHN2002401 12639229

[2] Food and Agriculture Organization of the United Nations (FAO). Sustainable diets and biodiversity. Directions and solutions for policy, research and action. *Proceedings of the International Scientific Symposium: “Biodiversity and sustainable diets against hunger”.* Rome: FAO (2012). Available online at: www.fao.org/docrep/016/i3004e/i3004e00.htm

[3] Tukker A Huppes G Guinée J Heijungs R Koning A Oers L Environmental Impact of Products (EIPRO): Analysis of the Life Cycle Environmental Impacts Related to the Final Consumption of the EU-25. *Institute for Prospective Technological Studies (IPTS), Joint Research Centre (JRC), European Commission, Seville.* Seville: European Commission (2006).

[4] Westhoek H Lesschen JP Rood T Wagner S De Marco A Murphy-Bokern D Food choices, health and environment: effects of cutting Europe’s meat and dairy intake. *Glob Environ Change.* (2014) 26:196–205. 10.1016/j.gloenvcha.2014.02.004

[5] Bailey R Harper DR. *Reviewing Interventions for Healthy and Sustainable Diets. Research Paper.* London: The Royal Institute of International Affairs (2015).

[6] Lang T. Sustainable diets: another hurdle or a better food future? *Development.* (2015) 57:240–56. 10.1057/dev.2014.73

[7] Springmann M Clark M Mason-D’Croz D Wiebe K Bodirsky BL Lassaletta L Options for keeping the food system within environmental limits. *Nature.* (2018) 562:519–25. 10.1038/s41586-018-0594-0 30305731

[8] El Bilali H Callenius C Strassner C Probst L. Food and nutrition security and sustainability transitions in food systems. *Food Energy Secur.* (2019) 8:e00154. 10.1002/fes3.15

[9] Willett W Rockström J Loken B Springmann M Lang T Vermeulen S Food in the anthropocene: the EAT-Lancet Commission on healthy diets from sustainable food systems. *Lancet.* (2019) 393:447–92. 10.1016/S0140-6736(18)31788-4 30660336

[10] Barilla Foundation & Research Unit on Nutrition, Diabetes and Metabolism, University of Naples Federico II. *A One Health Approach to Food, the Double Pyramid Connecting Food Culture, Health and Climate.* (2021).

[11] IPES-Food, ETC Group. *A Long Food Movement: Transforming Food Systems by 2045.* Brussels: IPES-Food (2021).

[12] HLPE. Food security and nutrition: building a global narrative towards 2030. *A Report by the High Level Panel of Experts on Food Security and Nutrition of the Committee on World Food Security, Rome.* Rome: HLPE (2020).

[13] Fanzo J. Healthy and sustainable diets and food systems: the key to achieving sustainable development goal 2? *Food Ethics.* (2019) 4:159–74. 10.1007/s41055-019-00052-6

[14] Meier T Gräfe K Senn F Sur P Stangl GI Dawczynski C Cardiovascular mortality attributable to dietary risk factors in 51 countries in the WHO European Region from 1990 to 2016: a systematic analysis of the Global Burden of Disease Study. *Euro. J. Epidemiol.* (2019) 34 37–55. 10.1007/s10654-018-0473-x PMC6325999 30547256

[15] Lang T Barling D. Food security and food sustainability: reformulating the debate. *Geograp J.* (2012) 178:313–26. 10.1111/j.1475-4959.2012.00480.x

[16] IPES-Food. Towards a common food policy for the EU- Framing paper for the EU food and farming forum 2018. *Presented at Eu Food and Farming Forum, 29-30 May 2018, Brussels.* Brussels: IPES-food (2018).

[17] Lawrence MA Friel S Wingrove K James SW Candy S. Formulating policy activities to promote healthy and sustainable diets. *Public Health Nutrit.* (2015) 18:2333–40. 10.1017/S1368980015002529 26282619 PMC10271405

[18] Aspenson A. *True Costs for Food System Reform: An Overview of True Cost Accounting Literature and Initiatives.* Baltimore, ML: Johns Hopkins Center for a Livable Future (2020).

[19] Sandhu H Gemmill-Herren B de Blaeij A van Dis R Baltussen W. Application of the TEEBAgriFood Framework: case studies for decision-makers. *TEEB for Agriculture & Food: Scientific and Economic Foundations.* Geneva: UN Environment (2018). p. 297–331.

[20] Global Alliance For Future of Food. *Accelerating True Cost Accounting.* (2021). Available online at: https://futureoffood.org/accelerating-true-cost-accounting/

[21] Irz X Leroy P Requillart V Soler LG. Welfare and sustainability effects of dietary recommendations. *Ecol Econ.* (2016) 130:139–55.

[22] Peters CJ Picardy J Darrouzet-Nardi AF Wilkins JL Griffin TS Fick GW. Carrying capacity of US agricultural land: ten diet scenarios. *Elementa Sci Anthropocene.* (2016) 4:116. 10.12952/journal.elementa.000116

[23] Conforti P D’Amicis A. What is the cost of a healthy diet in terms of achieving RDAs? *Public Health Nutr.* (2000) 3:367–73. 10979156 10.1017/s1368980000000410

[24] Donati M Menozzi D Zighetti C Rosi A Zinetti A Scazzina F. Towards a sustainable diet combining economic, environmental and nutritional objectives. *Appetite.* (2016) 106:48–57. 10.1016/j.appet.2016.02.151 26921487

[25] Alessandra D Maria M Cecilia C Augusto A Marcello V. The adherence of the diet to Mediterranean principle and its impacts on human and environmental health. *Int J Environ Prot Policy* (2014) 2:64–75.

[26] Germani A Vitiello V Giusti AM Pinto A Donini LM del Balzo V. Environmental and economic sustainability of the Mediterranean Diet. *Int J Food Sci Nutrit.* (2014) 65:1008–12. 10.3109/09637486.2014.945152 25088933

[27] Vitale M Giosuè A Vaccaro O Riccardi G. Recent trends in dietary habits of the Italian population: potential impact on health and the environment. *Nutrients.* (2021) 13:476. 10.3390/nu13020476 33572514 PMC7911362

[28] Barilla Foundation. *Italy and Food - Nutritional Challenges, Agriculture, Food Loss and Waste.* Austin, TX: (2019). Available online at: https://www.barillacfn.com/en/publications/italy-and-food/

[29] Riccardi G Giosuè A Calabrese I Vaccaro O. Dietary recommendations for prevention of atherosclerosis. *Cardiovasc Res.* (2022) 118:1188–204. 10.1093/cvr/cvab173 34229346

[30] Food Balance Sheets. *FAOSTAT.* Rome: FAO (2020).

[31] Petersson T Secondi L Magnani A Antonelli M Dembska K Valentini R A multilevel carbon and water footprint dataset of food commodities. *Sci Data.* (2021) 8:1–12. 10.1038/s41597-021-00909-8 33963181 PMC8105407

[32] Djuma H Bruggeman A Daskalakis D Hembury A Kozyra J Hammer J Irrigation water governance. *Enorasis Project Report.* Cyprus: The Cyprus Institute (2012). Available online at: http://www.enorasis.eu/uploads/files/D2%201_ENORASIS_GOVERNANCE_AND_BUSINESS_v12.pdf

[33] Panagiotakos DB Pitsavos C Stefanadis C. Dietary patterns: a Mediterranean diet score and its relation to clinical and biological markers of cardiovascular disease risk. *Nutr Metab Cardiovasc Dis.* (2006) 16:559–68. 10.1016/j.numecd.2005.08.006 17126772

[34] Panagiotakos DB Pitsavos C Arvaniti F Stefanadis C. Adherence to the Mediterranean food pattern predicts the prevalence of hypertension, hypercholesterolemia, diabetes and obesity, among healthy adults; the accuracy of the MedDietScore. *Prev Med.* (2007) 44:335–40. 10.1016/j.ypmed.2006.12.009 17350085

[35] Roggeri DP Roggeri A Rossi E Cinconze E De Rosa M Maggioni AP Direct healthcare costs and resource consumption after acute coronary syndrome: a real-life analysis of an Italian subpopulation. *Eur J Prevent Cardiol.* (2014) 21:1090–6. 10.1177/2047487313483608 23515447

[36] Bernaschi D Marino D. I Consumi. In: Marino D editor. *L’Atlante del Cibo Della Città Metropolitana di Roma Capitale. Uno Strumento per le Politiche Locali del Cibo.* Roma: CURSA (2021).

[37] Sandbag Smarter City Policy. *Carbon Price Viewer.* (2021) Available online at: https://sandbag.be/index.php/carbon-price-viewer/

[38] Cavaliere A De Marchi E Frola EN Bacenetti J Orlando F Banterle A. *Playing with the Mediterranean Diet: May Little Changes Benefit the Environment Without Compromising Health?.* Settembre Bologna: SIDEA Congress (2021).

[39] Duchin F. Sustainable consumption of food: a framework for analysing scenarios about changes in diets. *J Industr Ecol.* (2005) 9:99–114. 10.1162/1088198054084707

[40] Cevallos G Grimault J Bellassen V. *Domestic Carbon Standards in Europe Overview and Perspectives.* Paris: Institute for Climate Economics (2019).

[41] Donofrio S Maguire P Myers K Daley C Lin K. *State of the Voluntary Carbon Markets 2021.* Washington, DC: Ecosystem Marketplace (2021). Available online at: https://www.ecosystemmarketplace.com

