# Supplementary material for: True Cost Accounting of a healthy and sustainable diet in Italy
Source: Front Nutr. 2022 Jul 29;9:974768. doi: 10.3389/fnut.2022.974768 (PMC9372443; doi:10.3389/fnut.2022.974768)
Supplement: Supplementary file 1 [file Data_Sheet_1.docx]

**Supplementary materials**

**S.1. Explanation methodology**

**S.1.1. Water and Carbon footprint calculations:**

Based on Vitale et al. 2019, 15 food groups have been taken in consideration. For each food group, foods items have been selected along with their related carbon footprint and water footprint Sueatable values. Since the study needed data that were comparable among the impact dimensions considered (environment, health and socio-economic), the food items have been selected following the data availability of the different databases and their representativeness in the food group they belong to.

An average of food items’ carbon and water footprint values has been done when the food group portion didn’t refer to specific foods, while a sum has been done when the portions were more specifics (i.e., for recommended diet fruit portion is 400g/day while in milk and diaries, yogurt portion is 200g/day).

| **Food group** | **Average or sum** | **Food items** |
| --- | --- | --- |
| Fruit | average | Apple, pear, banana |
| Vegetables | average | Broccoli, eggplants, zucchini, lettuce |
| Legumes | single value | Dry beans |
| Nuts | single value | Almond without shell |
| Refined cereals | sum | Bread plain, pasta plain, cornflakes |
| Potatoes | single value | potatoes |
| Wholegrain | sum | Whole bread, whole pasta, whole crackers |
| Beef and pork meat | average | Beef meat with bone, pork meat with bone, beef meat without bone, pork meat without bone |
| Poultry | average | Chicken meat with bone, chicken meat without bone |
| Fish | sum | Anchovies, seabass |
| Eggs | single value | Eggs |
| Dairy products | sum | Yogurt, cow milk, ricotta, grana padano |
| Animal fat and tropical oils | single value | Olive oil |
| Non- tropical oils | sum | Beet sugar, honey |

Table S1: Food items selected for food groups on Petersson et al. 2021 database (source: Authors)

Each value of carbon (kg CO2 eq./kg of food) and water footprint (liters of water/kg of food) has been multiplied by the portion (g/day) (Table S2) and divided per 1000 (to convert kg into g).

For the desirable diet, the weekly amounts of foods have been provided by Riccardi et al., (2022) and are the following:

| **Food group** | **Amount (g/day)** |
| --- | --- |
| Fruit | 400 |
| Vegetables | 400 |
| Legumes | 46 |
| Nuts | 30 |
| Refined cereals | 94 |
| Potatoes | 50 |
| Wholegrain | 150 |
| Beef and pork meat | 14 |
| Poultry | 43 |
| Fish | 103 |
| Eggs | 43 |
| Dairy products | 329 |
| Animal fat and tropical oils | 3 |
| Non- tropical oils | 30 |
| Sugars | 20 |

Table S2: Amounts of food groups in the desirable diet based on the study by Riccardi et al. (2022)

For the current Italian diet**,** the weekly amounts of foods have been provided by the study by Vitale et al. (2021) and are:

| **Food group** | **Portion (g/day)** |
| --- | --- |
| Fruit | 181 |
| Vegetables | 205 |
| Legumes | 12 |
| Nuts | 18 |
| Refined cereals | 288 |
| Potatoes | 50 |
| Wholegrain | 27 |
| Beef and pork meat | 124 |
| Poultry | 41 |
| Fish | 57 |
| Eggs | 28 |
| Dairy products | 571 |
| Animal fat and tropical oils | 14 |
| Non- tropical oils | 46 |
| Sugars | 79 |

Table S3: Portions of current diet per food groups based on Vitale et al. 2021 (source: Authors)

Then, the obtained daily carbon and water footprint values for the food groups have been multiplied by 7 to achieve the weekly value.

S.1.1.1. **Water and Carbon costs**:

The weekly values of carbon and water footprint previously calculated have been multiplied by the carbon price (Carbon Price Viewer from Sandbag) and the cost of irrigation water (Djuma et al. 2012), respectively. The prices considered are:

- Carbon Price: 0,05 EUR/kg (as 50 EUR/ton)
- Water Price: 0,001 EUR/L (as the average between 1,16 EUR/m3 and 0,80 EUR/m3 highlighted by Djuma et al. 2012 to be the irrigation water range prices).

S.1.2. **Health impact**:

The health impact has been estimated by applying the Mediterranean Diet Score in the Italian population and evaluating the relationship between the diet score and the probability of having a CHD event during a 10-year period (Panagiotakos et al., 2006, 2007). In the study by Panagiotakos et al., 2007, the Score was used in the sample of the ATTICA study (Pitsavos et al., 2003) that enrolled 3042 participants from the Athens metropolitan area (during 2001–2002) to estimate the risk of CHD over the course of 10 years. It was allowed to apply in the Italian population the risk estimates supplied by this study, since low risk estimation charts should be considered for use both in Greece and Italy (Mach et al., 2019).

First, the frequency of consumption of the various food groups was scored for the current Italian diet (21) and for the desirable one (37). Higher values of the diet score indicate greater adherence to the Mediterranean diet (Panagiotakos et al., 2006, 2007). Second, the Diet Scores obtained for the current and the desirable diet were associated to the relative predicting 10-year CHD risks (%) in men and women aged from 41 to 70 years (adult Italian population) according to Panagiotakos et al., 2007.

A Mediterranean Diet Score of 21 is estimated to correspond to a 10-year risk of 5.4 %

A Mediterranean Diet Score of 37 is estimated to correspond to a 10-year risk of 4.3 %

Therefore, by implementing the recommended diet, the Italian population would experience a 21 % reduction in the incidence of CHD (myocardial infarction and other ischemic heart diseases).

The impact of the diet on Italian health system is then calculated with the use of Roggeri et al. 2014 data on treatment costs of coronary patients.

S.1.3. **Socio-economic impact**:

For each of the 15 food groups previously described (see section S1.1), the cost of single food items has been selected from the ISMEA database (not open access but accessible under request) with data from Totale Italia (DM + Discount) from 2018 to 2021. The database provides total volumes and economic values for a series of specific food items/groups. The same foods selected for the calculation of the carbon and water footprint of diets have been selected for this calculation (Table S.1). When one food was not present in the database, a similar item has been selected to proceed with the calculations. As Table S.5 shows the main differences are related to the fact that the ISMEA database doesn’t contain some specific food items but consider only the wider food group they belong to (Table S.5).

| **Food group** | **Items from Petersson et al. 2021** | **Items from ISMEA 2018-2021** |
| --- | --- | --- |
| Fruit | same | |
| Vegetables | Broccoli, eggplants, zucchini, lettuce | Carrots, eggplants, zucchini, lettuce, tomatoes |
| Legumes | Dry beans | Dry legumes |
| Nuts | Almond without shell | Nuts |
| Refined cereals | Bread plain, pasta plain, cornflakes | Packaged bread, pasta plain, breakfast cereals |
| Potatoes | same | |
| Wholegrain | Whole bread, whole pasta, whole crackers | Packaged whole bread, whole pasta, whole bread substitutes |
| Beef and pork meat | Beef meat with bone, pork meat with bone, beef meat without bone, pork meat without bone | Fresh meat pork and fresh meat beef |
| Poultry | Chicken meat with bone, chicken meat without bone | Fresh meat chicken |
| Fish | Anchovies, seabass | Fresh or frozen fish |
| Eggs | same | |
| Dairy products | Yogurt, cow milk, ricotta, grana padano | Yogurt, cow milk, fresh cheese, cured cheese |
| Animal fat and tropical oils | same | |
| Non- tropical oils | same | |
| Sugars | same | |

Table S.5: Comparison of food items selected from Petersson et al. 2021 and ISEM 2018-2021 databases (source: Authors)

For each food, an average of values from 2018 and 2021 has been calculated for both italian sales volumes and values, and the price per unit of food item has been then calculated by dividing total sales volumes and values. The economic value per kg or L of each food group has then been multiplied by the g/day portion value (see section S.1.1) and divided by 1000. Finally, the daily price has been multiplied by 7 to obtain the weekly value and by 4 to obtain the monthly value.

S.1.3.1. Affordability index:

Starting from the price of each food group (EUR), and affordability index has been calculated following Bernaschi and Marino, 2021:

- First, we calculated the incidence of food expenditure of the considered diets on income (%) dividing the total month expenditure of each diet (ISTAT, 2019) with the average net income declared (ISTAT, 2017). (A)
- Second, the average incidence of food expenditure on total consumption (%) has been obtained by dividing the national monthly food expenditure declared (excluding water, beverages and coffee) (ISTAT, 2020) with the monthly total expenditure declared (ISTAT, 2020). (B)
- Third, the average incidence of food expenditure on total consumption (B) has been divided by the incidence of food expenditure on income (A) obtaining the economic affordability index (C).

|  | **Desirable Diet** | **Current Diet** |
| --- | --- | --- |
| **B = INCIDENCE OF FOOD EXPENDITURE (+/-) ON INCOME** | 14% | 16% |
| **A = AVERAGE INCIDENCE OF FOOD EXPENDITURE ON TOTAL CONSUMPTION (ISTAT)** | 18,5% | 18,5% |
| **C = ECONOMIC ACCESSIBILITY INDEX (B / A)** | -24% | -11% |

Table S.6: Affordability index calculation (source: Authors)

The affordability index is a proxy of the impact of the diet on total income and food expenditure, namely the savings on the national food expenditure that the consumption of one diet could provide. Although ISTAT data on food expenditure refer to actual italian consumption data, the food basket used in this study does not totally correspond to the food basket used by ISTAT. For instance, water, beverages, and coffee have been eliminated along with ready meals. The reason of this difference is related to the methodology of this study which created an ad hoc food basket that would be common to all calculations (environmental, health and economic). Hence, ISTAT data have been used as reference data and the value of this affordability index relies in the comparison between the desirable and the current Italian diet rather than between ISTAT and current diet data.
